# Ligand and structure-based virtual screening applied to the SARS-CoV-2 main protease: an *in silico* repurposing study

**DOI:** 10.4155/fmc-2020-0165

**Published:** 2020-08-13

**Authors:** Witor Ribeiro Ferraz, Renan Augusto Gomes, Andre Luis S Novaes, Gustavo Henrique Goulart Trossini

**Affiliations:** ^1^Faculdade de Ciências Farmacêuticas, Universidade de São Paulo; Av. Prof. Lineu Prestes, 580, São Paulo, SP, 05508-000, Brasil

## Abstract

**Aim:** The identification of drugs for the coronavirus disease-19 pandemic remains urgent. In this manner, drug repurposing is a suitable strategy, saving resources and time normally spent during regular drug discovery frameworks. Essential for viral replication, the main protease has been explored as a promising target for the drug discovery process. **Materials & methods:** Our virtual screening pipeline relies on the known 3D properties of noncovalent ligands and features of crystalized complexes, applying consensus analyses in each step. **Results:** Two oral (bedaquiline and glibenclamide) and one buccal drug (miconazole) presented 3D similarity to known ligands, reasonable predicted binding modes and micromolar predicted binding affinity values. **Conclusion:** We identified three approved drugs as promising inhibitors of the main viral protease and suggested design insights for future studies for development of novel selective inhibitors.

## Graphical abstract

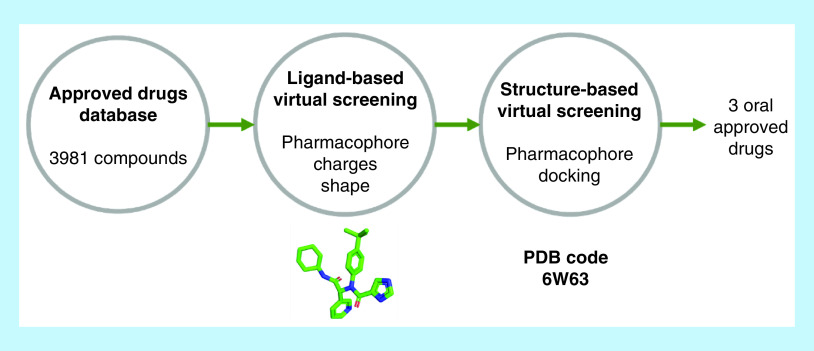

The pandemic outbreak of the SARS-CoV-2 (severe acute respiratory syndrome-related coronavirus-2) infection, known as COVID-19 (coronavirus disease 2019), has become a worldwide public health emergency. By May of 2020, over 4.5 million infected people were reported and 310000 had lost their lives [1]. Heavily affected regions are reporting improvements on new infections and deaths. Nevertheless, these improvements are based on severe social isolation and lockdowns, causing huge social and economic impacts, which may compromise healthcare systems [2]. Important epidemiological characteristics, such as the case fatality rate (CFR), infectiousness start and duration, number of asymptomatic cases, risk of reactivation or reinfection [3], are still unknown. Those open questions are alarmingly pointing to the possibility of future reinfection waves [4].

In face of the urgent circumstances, drug repurposing has become the main strategy for the scientific community, since pharmacokinetic and toxicological data are available, speeding up clinical trials and requiring less time and investment to bring the drug to market [5]. For instance, the US FDA has launched the Coronavirus Treatment Acceleration Program (CTAP) [6] and specific guidance on COVID-19 clinical trials [7]. Additionally, the FDA has issued emergency use authorizations for some drugs, for example, chloroquine, hydroxychloroquine and remdesivir [8,9]. Several papers have been published focusing on repurposing drugs for the treatment of COVID-19 [10], some of which might be more promising after preliminary clinical reports. Chloroquine and hydroxychloroquine, common antimalarial drugs, have been reported to have *in vitro* activity against diverse RNA viruses, including SARS-CoV-2 [11]. They may act through multiple mechanisms such as the inhibition of viral entry and release from host cells, reduced viral infectivity and host immune modulation properties [12]. However, recent clinical trials investigating this drug concluded that the treatment was not significantly correlated to intubation risk or mortality by multiple analyses [13,14]. Remdesivir, a nucleotide prodrug, inhibits viral RNA-dependent RNA polymerase and has been reported to have *in vitro* activity against SARS-CoV-2 [15]. Ivermectin, a broad spectrum anti-parasitic agent, inhibits replication of SARS-CoV-2 by blocking viral proteins from entering the host cell's nucleus, keeping the host antiviral response intact [16]. Macrolide antibiotics have been reported to be beneficial to the immunomodulation of chronic pulmonary disorders [17]. The combination of azithromycin and hydroxychloroquine has also been administered for the treatment of COVID-19 patients [18]. Heparin, an anticoagulant agent, inhibits thrombotic phenomena and has been reported to improve hypoxia in severely ill COVID-19 patients [19]. All these drugs still lack evidence of therapeutic efficacy and many others are in preclinical and clinical trials.

Several structural components of the coronavirus family viruses have been described as biotargets for drug discovery. These targets are related to viral nucleic acids, enzymes, spike glycoprotein and envelope (membrane, nucleocapsid and accessory proteins) [20,21]. A massive effort has been undertaken by the international scientific community to obtain structural information about complexes between SARS-Cov-2 targets and different types of inhibitors, providing information to use ligand-based (LB) and structure-based (SB) approaches to drug repurposing. The present work evaluated the druggability of the SARS-CoV-2 main protease (SARS-CoV-2 M^pro^) as a potential target for approved drug for the treatment of COVID-19 as it plays a crucial role in the cleavage of viral polyproteins involved in transcription and replication. This enzyme is reported to be inhibited by several classes of compounds, some of which exhibit anti-CoV activities *in vitro*, *in vivo* and even in nonrandomized trials (such as lopinavir) [22]. Similar to our work, other virtual screening campaigns have been conducted to identify candidate inhibitors of the same target. All of them were SB studies, unanimously employing molecular docking as main filtering step. The major difference among them was the dataset to be screened: two phytochemical sets (38 [23] and 65 [24] compounds), an FDA-approved antiviral set (124 compounds) [25] and a ZINC library (1.3 billion compounds) [26]. We believe our study contributes in different ways to previous research about target inhibition by approved drugs [27,28]. Firstly, our dataset comprised approved drugs from the FDA and the EMA. Second, our SB analysis considered a crystal of M^pro^ in complex with a competitive noncovalent lead-like inhibitor, a compound of similar size, complexity and mechanism of inhibition to the drugs to be screened. Furthermore, we employed a hierarchical virtual screening framework, which relied on the shape, electrostatic and chemical features of ligands in crystalized complex with M^pro^, and a SB pharmacophore model and molecular docking. Of 3981 drugs, ten were found to be promising; three were defined as the most likely according to our strategy based on consensus analysis.

## Materials & methods

### Database construction

For our repurposing study, 3981 approved drugs were used for 3D database construction. They were first submitted to QUACPAC 2.0.2.2 (OpenEye) [29] for the determination of protonation state at physiological pH and for atomic charge calculations employing the AM1BCC semiempirical methodology [30]. Then, OMEGA 2.5.1.4 (OpenEye) [31] was used for the exploration of conformational space and conformer generation for each drug [32].

### LB virtual screening

Currently available SARS-CoV-2 main protease structures in the Protein Data Bank (PDB) were analyzed using Discovery Studio Visualizer (Dassault Systèmes BIOVIA, CA, USA) [33]. They were grouped according to the presence or not of inhibitors and their classification (i.e., drug-like inhibitor or molecular fragment, orthosteric or allosteric binding, and covalent or noncovalent inhibition). Up to the date of this work, the only available structure in PDB of the SARS-CoV-2 main protease in complex with a noncovalent inhibitor was with X77, a potent broad-spectrum noncovalent inhibitor (PDB code 6W63, resolution of 2.10 Å). Second, LB pharmacophore modeling was performed employing ROCS (OpenEye)34 for the similarity search based on 3D shape and chemical features [34]. Of the best 500 ranked compounds, EON (OpenEye)35 was used for the electrostatic potential similarity search. X77 was used as the query and positive control for both of them [35]. Additionally, other available ligands in complexes with M^pro^ on PDB were compared with the compounds selected by our model since there is not enough available endpoint data (IC_50_ and/or K_i_) for noncovalent M^pro^ inhibitors.

### SB virtual screening

The SB pharmacophore modeling was generated by the CavityPlus [36] web server using the complex of SARS CoV-2 M^pro^ and X77 as the query (PDB code 6W63, resolution of 2.10 Å). To perform the molecular docking, the target structure from the same crystal was prepared using the protein preparation tool in Chimera 1.12. Then, the selected drugs from ligand-based virtual screening (LBVS) were docked using DockThor (National Laboratory of Scientific Computation, LNCC, Petrópolis, Brazil) [37] and Autodock Vina (the Scripps Research Institute, CA, USA) [38] at default settings using a 15 Å grid box centered at x = -20.313 y = 18.742 z = -28.341. The score functions of both programs rely on binding affinity, in kcal/mol, providing more realistic information regarding potency. However, they differ in the calculation methodology. Autodock Vina considers inter and intramolecular interactions (i.e., hydrophobic interactions and hydrogen bonding) for the enthalpic term and the rotatable bonds between heavy atoms in the ligand for the entropic term [38], while DockThor uses the MMFF94S force field to calculate those intra and intermolecular potentials, also considering the torsional term [39]. Binding affinity values were analyzed using Student's unpaired t-test with Welch's correction with GraphPad Prism 7 software (GraphPad Software Inc.) [40]. For further investigation, the best docking poses were compared with other complexes available in the PDB.

## Results & discussion

In this repurposing work, a set of EMA and FDA-approved drugs was submitted to two-step hierarchical virtual screening employing LB and SB approaches to identify drugs to target the SARS-CoV-2 main protease. First, the processed database was screened by two different LB approaches, ROCS and EON, which are very accurate methods based on molecular shape and electrostatically profile, respectively [41]. Then, the LB hits were further evaluated using two different SB approaches, AutoDock Vina (Broyden–Fletcher–Goldfarb–Shanno algorithm) and DockThor (genetic algorithm) for molecular docking, and CavityPlus SB pharmacophore modeling for docking pose interpretation. In order to enhance the success rate of this virtual screening (VS) campaign, 2D approaches were discarded due to their intrinsic limitations and 3D methods were employed since they are more sensible and retain information [42]. Furthermore, a consensus analysis was performed in each step (LB and SB) to select the most promising compounds [43].

### Structure selection of the main protease for pharmacophore modeling

**Figure 1.**
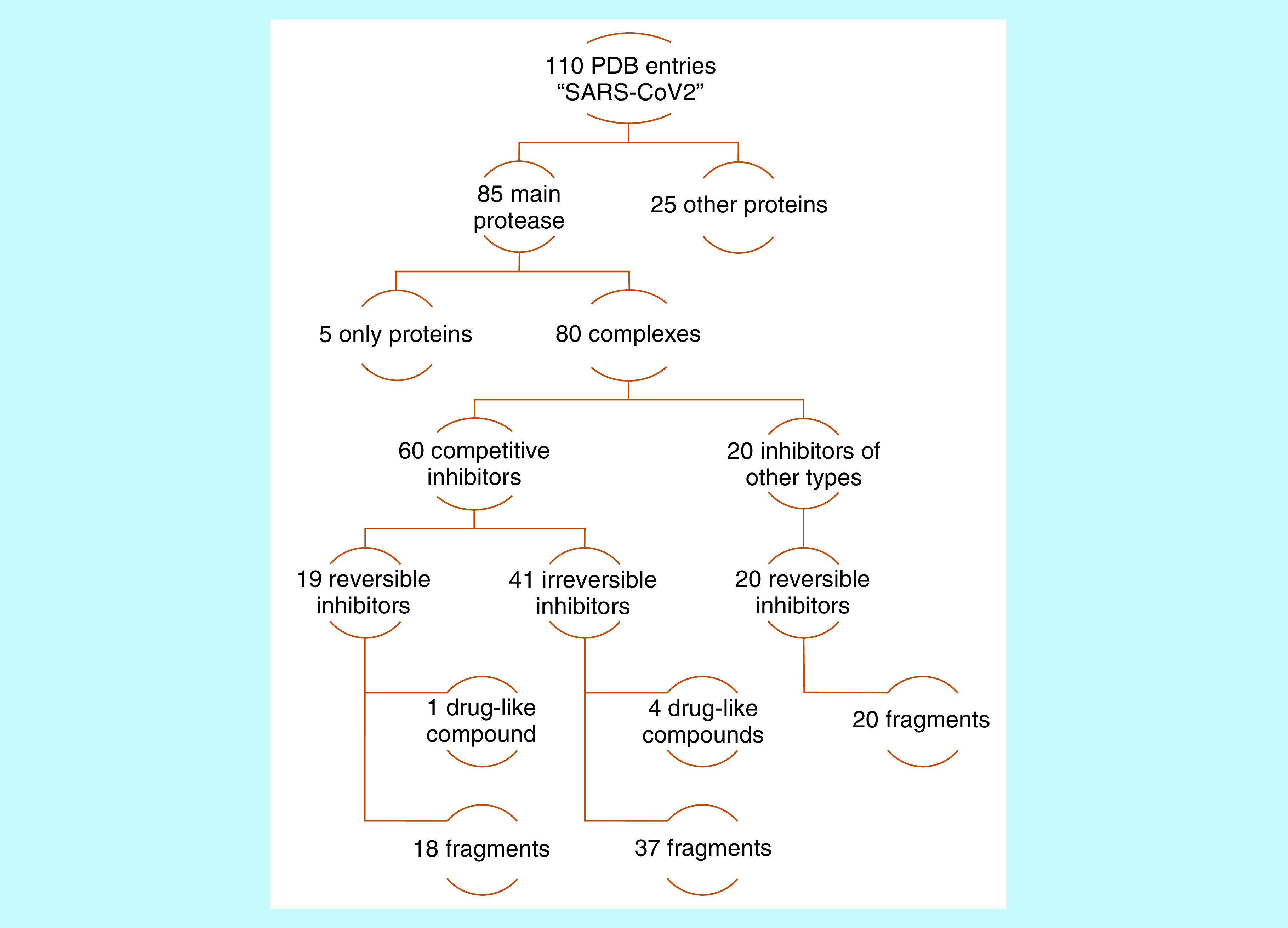
Analysis of 100 available SARS-CoV-2 structures in Protein Data Bank. Protein Data Bank entries were binarily classified according to protein name, being in complex or not, orthosteric binding site occupation or not, covalent inhibition or not, and ligand size.

From 110 SARS-CoV-2 proteins available in PDB at the moment of this work, 85 of them were the SARS-CoV-2 main protease, evidencing how important this target is in the development of coronavirus treatments. Analyzing them (Figure 1), five free proteins were found and 80 complexes, including 60 complexes with small molecules in the orthosteric binding site and 20 at different sites on the protein surface. For inhibitor classification, it was also considered if they bound covalently or not M^pro^ and if the size corresponded to molecular fragments or a drug-like compound. The majority of these competitive inhibitors (41 of 60) were α-ketoamides and α-ketosulphonamides, which bound covalently to the CYS145 sidechain, and only 19 noncovalent inhibitors were identified (one drug-like and 18 molecular fragments). For the purpose of our study, these 19 competitive noncovalent inhibitors were considered for pharmacophore modeling and docking pose evaluation.

**Figure 2.**
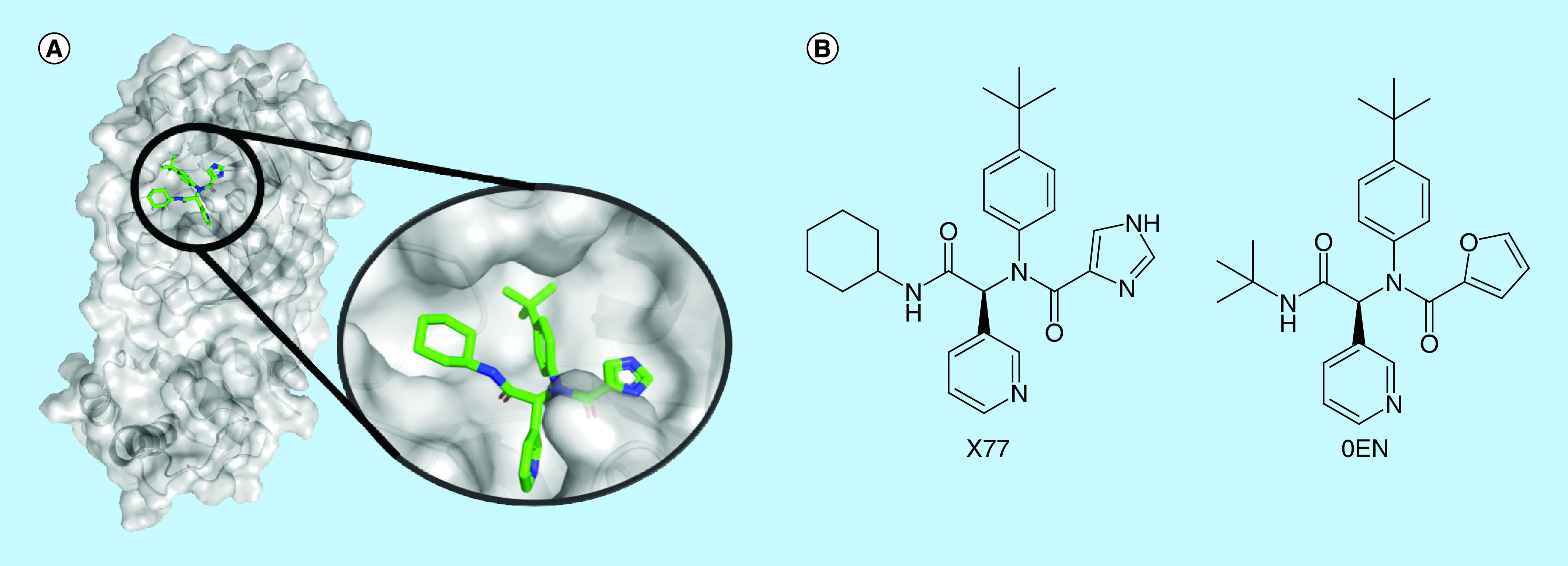
Complex of the SARS-CoV-2 main protease and X77. **(A)** SARS-CoV-2 main protease in complex with X77 inhibitor and the ligand in evidence. Ligand represented as sticks (carbons in green, nitrogens in blue and oxygens in red) and protein as cartoon and surface. **(B)** X77 and 0EN 2D structures, noncovalent inhibitors of main protease of SARS-CoV-2 and SARS-CoV, respectively.

To consider the induced fit in the different subareas of the binding site, the complex formed by the SARS CoV-2 main protease and the noncovalent inhibitor X77 (Figure 2) was chosen as the query for SB modeling. Although X77 is a new compound, there are findings that support its choice as a template for this study. In 2013, Jacobs and associates [44], as a result of a high-throughput screening campaign, discovered a hit to target SARS-CoV M^pro^. One of the main achievements during the hit to lead phase was a noncovalent inhibitor analogous to X77, N-[(1R)-2-(tert-butylamino)-2-oxo-1-(pyridin-3-yl)ethyl]-N-(4-tert-butylphenyl) furan-2-carboxamide, also known as 0EN. The R isomer of 0EN had an IC_50_ of 1.5 ± 0.3 μM and K_i_ of 1.6 ± 0.26 μM and is available in complex with the SARS-CoV main protease in the PDB (PDB code 3V3M). Although the complexes including fragments were very similar to this one, the side chain perturbation provoked by fragment complexation was localized due to their small size. Since our aim was to find an approved drug that would be able to perform as many noncovalent interactions as possible with different areas of this protease, X77 was the only cocrystalized ligand that matched the optimal drug size and complexity for this purpose. Thus, the information about X77 analog and the analysis of available noncovalent SARS-CoV-2 M^pro^ inhibitors provided us good evidence for using X77 as the query for LB pharmacophore modeling.

### LB virtual screening

The 40 highest ranked compounds, equivalent to one percent of the initial approved drug database, were considered hits according to ROCS and EON score functions, Tanimoto Combo and ET Combo, respectively. Only one compound was present in the top 1% of both rankings, in other words, niaprazine, a sedative-hypnotic drug. Also, there were some other remarkable results: two classes of drugs had outstanding rankings, the antidiabetic sulphonylureas (tolazamide, glibenclamide and gliclazide) were in the best ten ranked compounds by ROCS, the antifungal azoles (miconazole, econazole, sulconazole, tioconazole and efinaconazole) were in the ROCS and EON hit list, and bedaquiline, a antitubercular drug, presented remarkable shape similarity to X77 and was present in the EON hit list.

**Figure 3.**
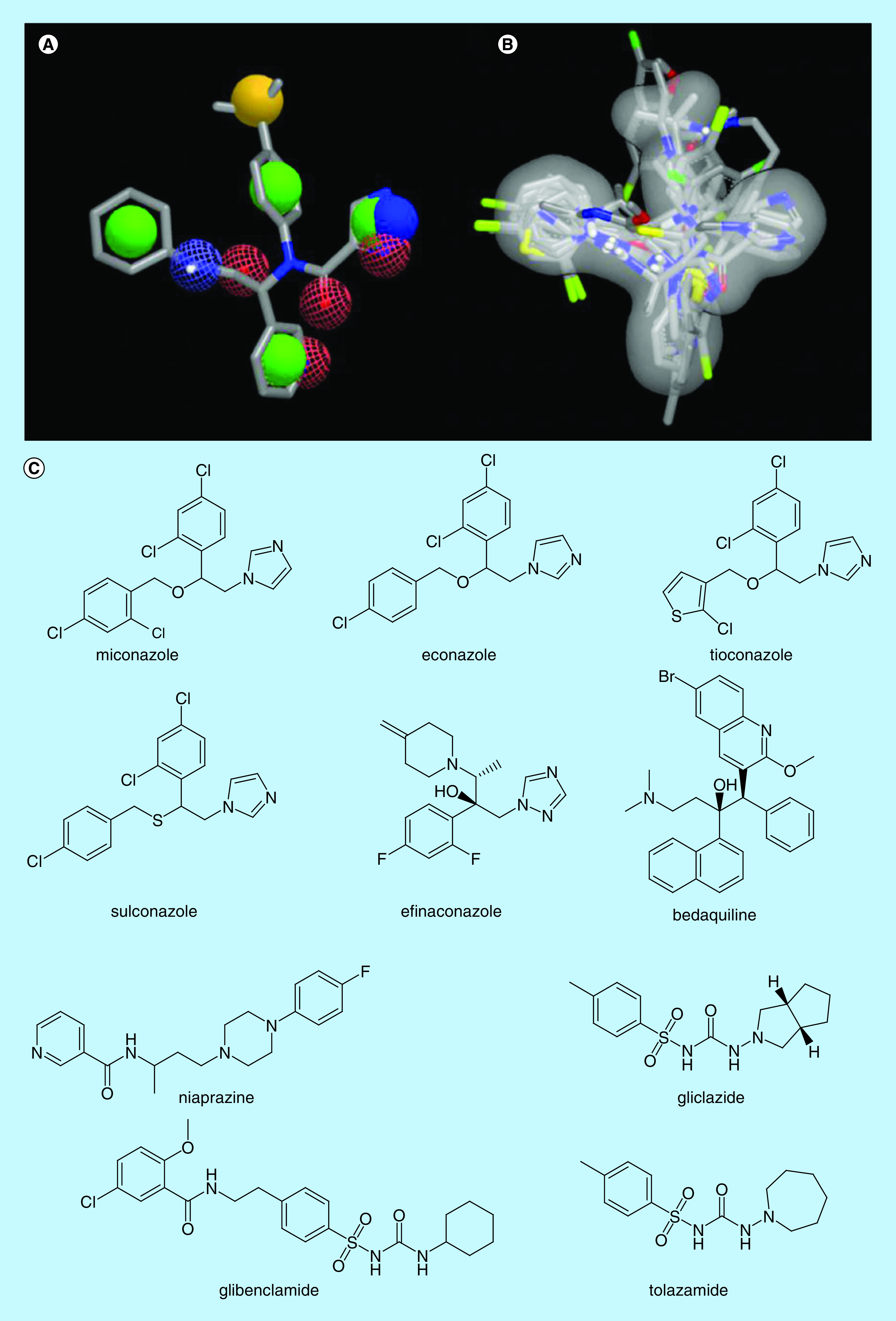
The X77 structure with highlighted features that were used by ROCS for virtual screening. **(A)** In solid green sphere, rings; in solid yellow, hydrophobic groups; in solid blue, cationic groups; in mesh blue, hydrogen bonds donor groups; and in mesh red, hydrogen bonds acceptor groups. **(B)** All the selected hits were aligned over the X77 molecular shape (surface in grey) and their **(C)** 2D chemical structure.

Amide (PDB code 5RF7 [45], 5R7Z [46], 5RE4 [47] and 5REZ [48]), sulphonamide (PDB code 5RF1 [49] and 5R80 [50]) and urea (PDB code 5R83 [51] and 5R84 [52]) moieties were found in several fragments in complex with the main protease, supporting the selection of antidiabetic drugs. Nitrogen heterocycles also were found, and lipophilic rings such as pyridine (PDB codes 5RE4 [47], 5REH [53], 5RF6 [54], 5R82 [55], 5R83 [51] and 5R84 [52]) and 1,2,3,4-tetrahydroquinoline (PDB code 5R81 [56]) were observed in the majority of cases, in agreement with niaprazine and bedaquiline. Also, very hydrophobic groups were identified, such as 4-bromo-phenyl and 5-fluoro-indol-3-yl (PDB codes 5RF1 [49] and 5R7Z [46], respectively), in consonance with all chosen drugs, but especially with bedaquiline and azoles. Hence, these ten compounds had the necessary features (Figure 3) and were selected for the SB step of this VS.

### SB virtual screening

Pharmacophore modeling performed by CavityPlus was carried out to identify the most important groups (in the backbone and/or sidechain) in the target to be considered for molecular binding with the different drugs, since it is already known in which pocket catalysis occurs. The algorithm identified nine features: two hydrophobic, two HBD, two HBA and three HBA combined with HBD (hybrid feature). Adopting the subdivision of the binding site made by Zhang and associates [57] with four different regions (S1–S4), these pharmacophore features were analyzed according to nearby residues present in each binding site subdivision, as described below.

In S1, the HBD feature was located in a highly exposed to solvent area according to our analysis in Discovery Studio Visualizer. It was not surprising to find water molecules in different cocrystalized structures creating hydrogen bonds with the N142 sidechain. Due to this fact, this feature was rejected. Also, in S1, one hydrophobic and one HBA combined with HBD features were close to each other. They all were kept because many ligands in the analyzed complexes present nitrogen heterocycles and this area was classified as being poorly accessible by the solvent and slightly hydrophobic.

**Figure 4.**
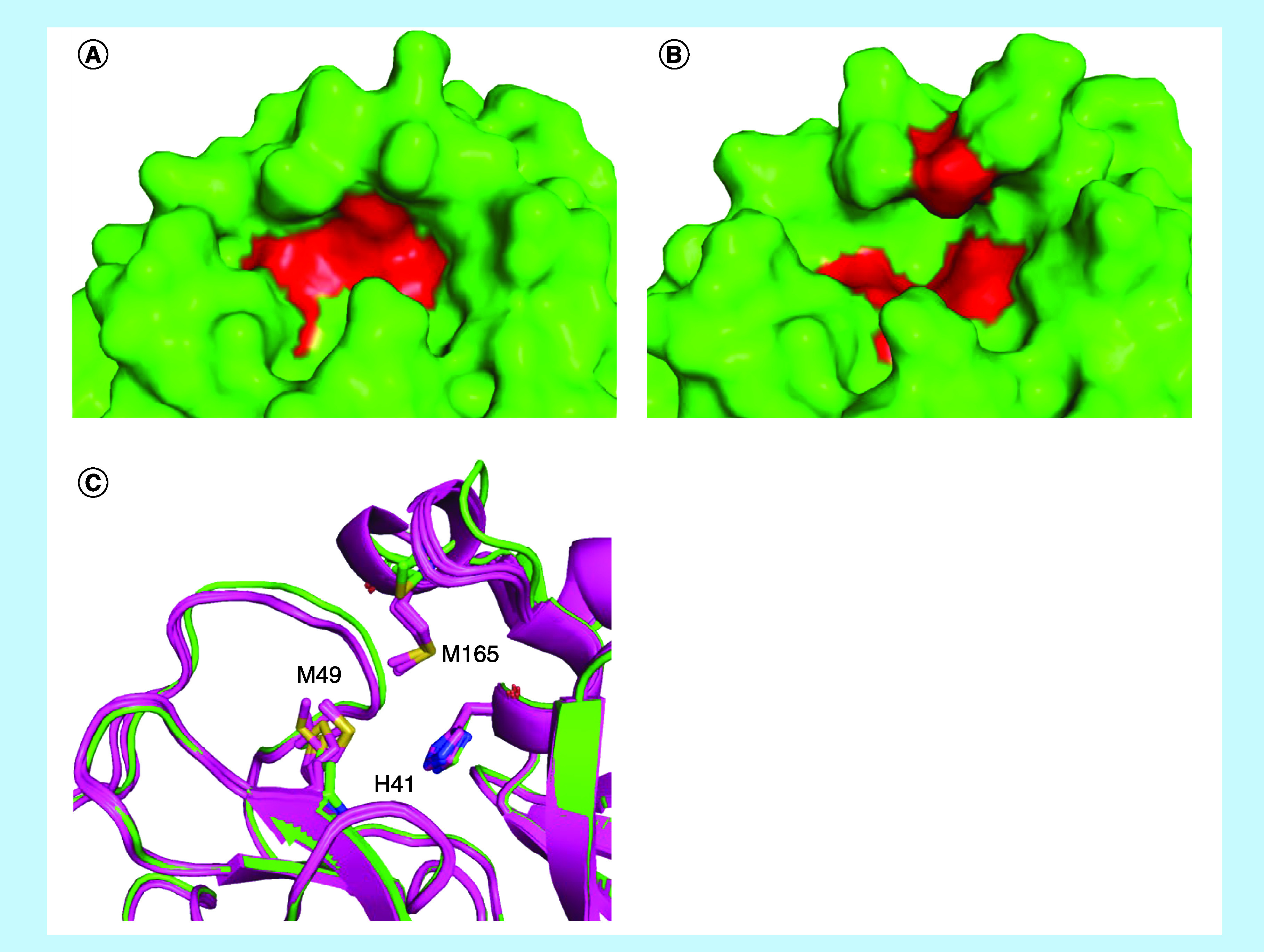
Conformational analyses of the SARS-CoV-2 main protease S2 pocket. **(A & B)** Conformational comparison of S2 pocket depth in complex, the main residues (H41, M49 and M165) are highlighted in red. **(C)** Comparing before (PDB code 6M03, 5R8T, 6Y84, 6YB7 and 6Y2E, all of them in magenta) and after (PDB code 6W63 in green) the complex formation, H41 remained with no remarkable modifications, M165 occupied the same region even with some variation, and M49 sidechain adopted one preferred conformation for the protein and complexes with fragments interacting with other portions of the binding site, and another conformation for the structure of M^pro^ in complex with X77, which interacted with this pocket. Protein structures were represented as cartoons and side chains as sticks. PDB: Protein Data Bank.

In S2, there were no crucial features identified by the CavityPlus algorithm. However, this region of the cavity suffered significant expansion due to a conformational change in complexation (Figure 4). There are three main residues in S2, H41, M49 and M165. The most remarkable change is related to the M49 side chain, which presented one preferred conformation for the protein (PDB code 5R8T [58], 6M03 [59], 6Y2E [57], 6Y84 [60] and 6YB7 [61]) and complexes with fragments that interacted with other portions of the binding site (PDB code 5R7Y [62], 5R7Z [46], 5R83 [51], 5RE4 [47], 5REH [53], 5REZ [48], 5RF1-3 [49,63,64], 5RF6 [54] and 5RFE [65]). When inhibitors were closer to M49, no pattern was observed, suggesting the mobility of this sidechain during the binding event. After this induced change, S2 became deeper and less accessible to the solvent, classified by Discovery Studio Visualizer, and no structural solvent molecules were found during the visual inspection. Furthermore, M49, H41 and M165 were identified in the greater part of the analyzed complexes performing hydrophobic interactions, which led us to add a hydrophobic feature in the SB-pharmacophore model.

In S3, only an HBA feature was spotted and not coincidentally many water and dimethyl sulfoxide (DMSO) molecules, suggested in the crystal structures, could be identified in this exposed hydrophilic region in the majority of the crystalized structures interacting with the T190 carbonyl and Q192 carbamoyl groups. Since no ligands dislocated the solvent, this feature was not considered to constitute the pharmacophore model.

In S4, an HBA and an HBD were identified. The first one was placed close to the Q189 carbamoyl group, so exposed to the solvent that a ligand displaced it to create a hydrogen bond in just one complex (PDB code 5R82). For the second feature, which was close to the carbonyl group at D166, water molecules and ligands were identified creating hydrogen bonds in a balanced frequency. Considering that the solvent was substantially displaced to interact with D166 and not Q189, it was decided to keep only the HBD.

**Figure 5.**
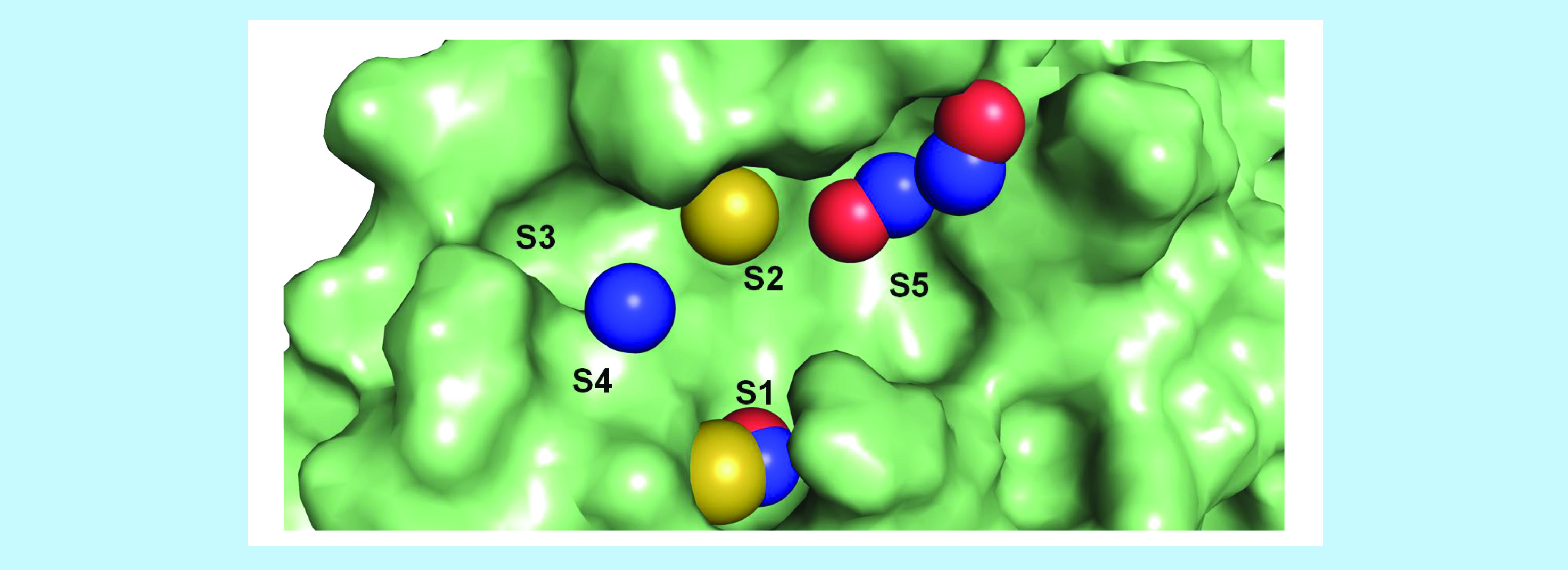
Representation of structure-based pharmacophore model based on SARS-CoV-2 main protease structure and its five subdivisions (S1cS5). Pharmacophore model features: yellow – hydrophobic, blue – hydrogen bond donor, red – hydrogen bond acceptor.

Additionally, a fifth region in the orthosteric binding site (henceforth called S5) was analyzed by the algorithm: one hydrophobic and two HBA combined with HBD features were placed in it. The first one was located very close to C145, the main catalytic residue of this cysteine protease [57,66], and L27. However, water molecules and HBA/HBD were found in many complexes near this subregion due to donors and acceptors in T26 (peptidic carbonyl), H41 (imidazole ring) and G143 (peptidic amino). In the superior part of S5, two HBA combined with HBD features were placed among several polar groups: T25 (hydroxyl), H41 (peptidic carbonyl and imidazole ring) and C44 (peptidic carbonyl). Not easily accessed by the solvent, different ligands were capable of dislocating water molecules in many crystals. Thus, the HBA/HBD features were kept while the hydrophobic one was removed. Figure 5 summarises these nine remaining features on the five subareas in the binding site.

**Figure 6.**
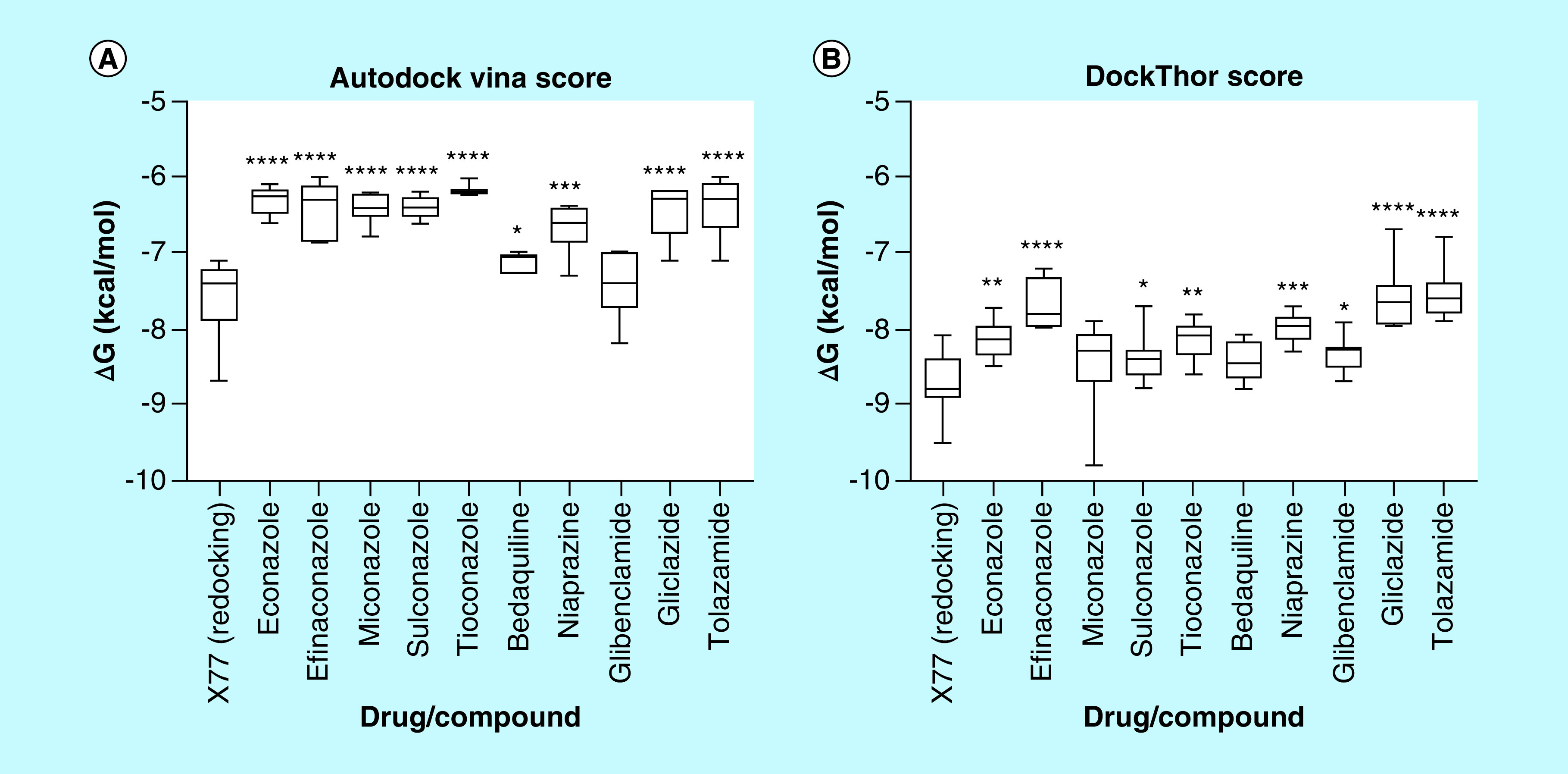
Predicted binding affinity values by Autodock Vina and DockThor algorithms for X77 redocking (PDB code 6W63) and docking screening for ten approved drugs. PDB: Protein Data Bank.

Once the SB pharmacophore model was generated and analyzed, the redocking of X77 was performed for evaluating pose prediction success and score function for both docking algorithms. Despite score functions in docking algorithms to deal with some problems and to reproduce experimental results in different scenarios, the Autodock Vina (AV) and DockThor (DT) best solutions (i.e., lower binding affinity) presented the lowest root mean squared deviation (RMSD) values (lower than 2.0 Å), and the interactions found in the crystalized complex were consistently reproduced, which allowed us to continue. Although both units of both score functions are expressed in kcal/mol, their respective equations consider different terms, making the comparison of values obtained from different methods not trustworthy. As the benchmark for our virtual screening procedure, the binding affinity values of every pose for each compound were compared with the values of X77 using Student's unpaired t-test (Figure 6). This approach was adopted because, when docking nonnative molecules into crystalized structures, all poses or only the best ranked should be evaluated to avoid bias.

**Figure 7.**
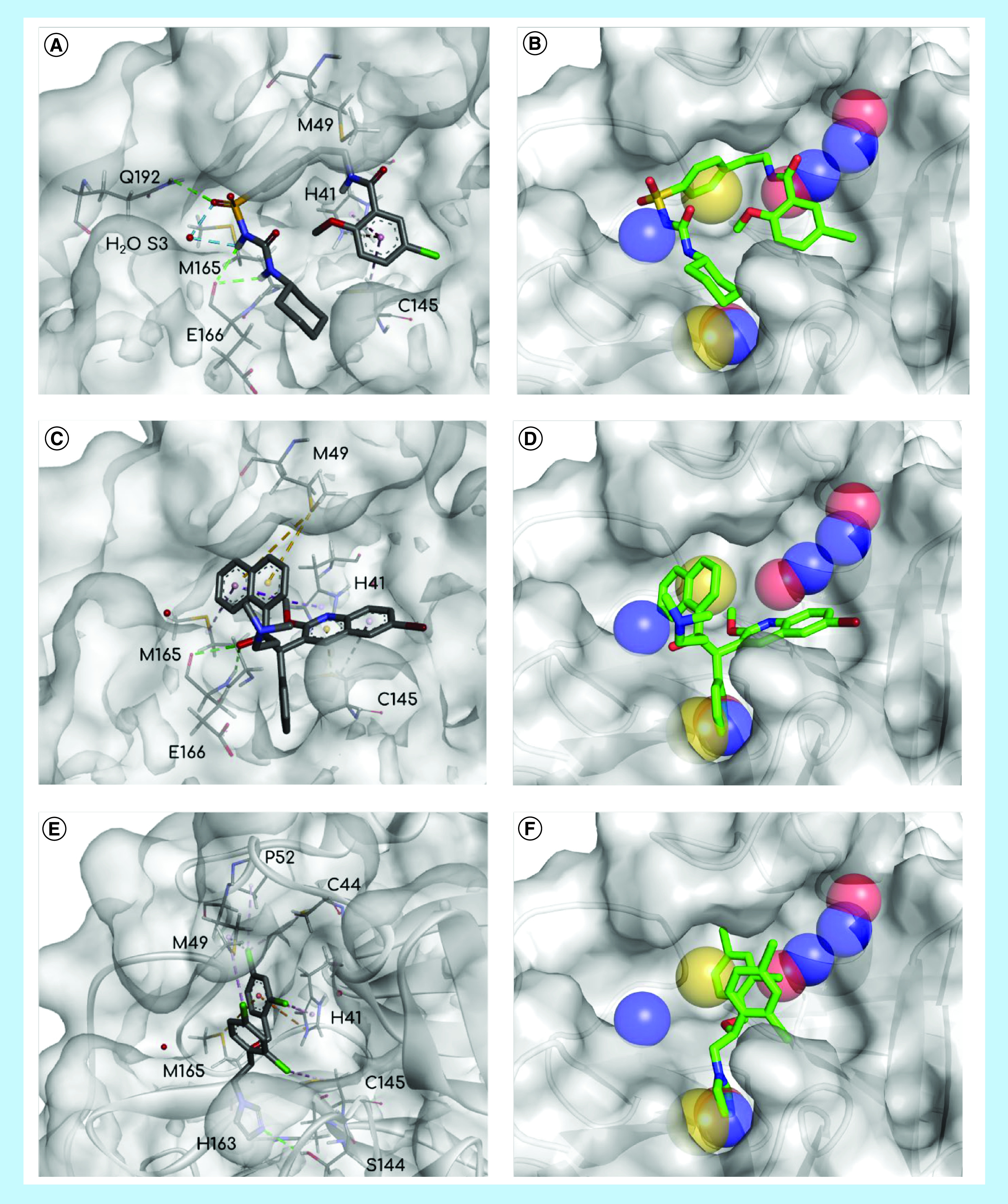
Docking of the selected hits and SARS-CoV-2 main protease. Poses of the three hits, glibenclamide **(A & B)**, bedaquiline **(C & D)** and miconazole **(E & F)**, interacting with SARS-CoV-2 main protease (figures on the left) and juxtaposed with SB pharmacophore model (figures on the right). Pharmacophore model features: yellow – hydrophobic, blue – hydrogen bond donor, red – hydrogen bond acceptor. Interactions: blue or green – hydrogen bonds, orange – π-cation, purple – nonpolar interactions. SB: Structure based.

Due to their linear shape and rotatable bonds, antidiabetic sulphonylureas did not fit into all subregions of the M^pro^ pocket or most of the pharmacophore features, providing some conformational freedom for both docking algorithms to generate poses. In this way, many docking solutions (generated by AV and DT) occupied S2, S3 and S5, establishing hydrogen bonds between sulphonylurea moiety and E166, as expected by the SB pharmacophore. However, the distal predominantly nonpolar groups of this class were not convergent regarding RMSD in relation to each other nor interacting with those expected hydrophobic residues, especially in S2, even being exposed to the solvent in some cases, reducing the reliability of these poses. As a consequence of this lack of complementarity, AD and DT binding affinity values were lower than the VS mean for gliclazide and tolazamide. For glibenclamide, likely as a result of some its more complex structure, some poses were more coincident to the CavityPlus pharmacophore and interacted with H41, M49, M165 at S2, a water molecule at S3, E166 at S4 and C145 at S5 (Figure 7A), as expected for a good inhibitor, which justified its closer binding energies to X77 than predicted by DT. In summary, the different docking solutions for gliclazide and tolazamide performed polar interactions with the main protease, but their nonpolar groups were noticeably loose in the binding site, which suggests that these compounds may inhibit the target but not in a potent way; however, for glibenclamide, some poses suggested its interaction may be strong as that of X77.

Regarding the antidiabetic drugs, niaprazine did not completely occupy the M^pro^ binding site. Because of its similar linear shape and relatively flexible structure, U-shaped poses were frequently observed, blocking only S2 and S5. On the other hand, this drug's docking solutions were consistently performing nonpolar interactions with H41, M49 and M165. When comparing solutions from AV and DT, AV converged more often to the SB pharmacophore HBD/HBA features in S5. However, regarding binding affinity, both AV and DT values were considerably distant from those of X77.

Bedaquiline performed better than the previous drugs since the format of its scaffold is analogous to a cross, allowing this molecule to fit into more pharmacophore features. Because of its many aromatic groups, the expected nonpolar interactions with H41, M49 and M165 in S2, C145 in S5 and the hydrophobic feature in S1 were repeatedly satisfied. Another common characteristic was the intramolecular interactions between nonpolar (π systems and alkyl groups) and polar (hydroxyl and methoxy substituents), favoring pose stabilization (Figure 7B). These combined enthalpic and entropic factors may explain the binding affinity for this drug being close to that of X77 and the predicted energy by DT statistically equivalent to it.

By virtue of their convenient properties, azole poses were the most complementary in this docking study. The nonlinear format and the higher number of rotatable bonds in the core of the scaffold provided extra conformational freedom for them to fit into the binding site when compared with the previously analyzed compounds. As a result of halogen-substituted phenyl groups and a deficiency in polar groups, the known lipophilic profile of this class may have contributed to different drugs performing correspondent interactions. The hydrophobic S2 region was consistently occupied with a substituted phenyl ring (Figure 7C). As a polyvalent group, the five membered heterocycle (imidazole, triazole or thiazole) created hydrogen bonds as a donor or acceptor in S1 and S5, fitting into the hybrid pharmacophore features, and π interactions with cysteine and histidine residues. Also, all the three branches of these drugs were attached to the binding site in the majority of the poses and rarely exposed to the solvent. Predominant nonpolar interactions and a lack of conformer stabilization by intramolecular forces may justify the weaker predicted binding energies for this drug class. However, some binding affinity values for miconazole were even stronger than those predicted for X77, highlighting this compound.

**Figure 8.**
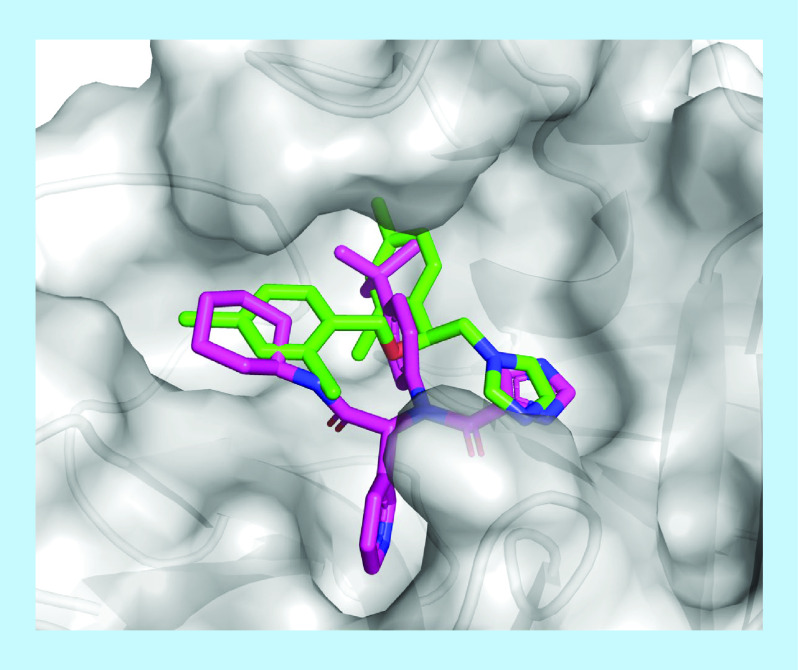
Miconazole (green) docking solution and X77 (magenta) in complex with SARS-CoV-2 main protease.

Comparing the poses of these three hits, glibenclamide, bedaquiline and miconazole, with fragments in complex with the main proteases, some similarities were found. In S2, several hydrophobic groups (e.g., phenyl [51,62,65], cyclohexyl [52] and thiophenyl [48,67]) were found interacting with M49, H41 and M165 via Van der Waals interactions, as well as π-π stacking/T-shaped interactions with H41 if the group was aromatic. The nonpolar groups of miconazole and bedaquiline, in other words, 2,4-dichloro-phenyl and naphthyl, respectively, interacted consistently with these residues. As common as nonpolar interactions in S2, amide, sulphonyl and urea moieties of many fragments created hydrogen bonds with E166 in S3 in many structures [45–52], in the same position of glibenclamide docking solutions. The last recognized pattern in PDB structures was aromatic groups with HBA (five of them were pyridinyl groups) or DMSO molecules. The nitrogen atom was in position 3 and buried in the pocket and the oxygen atom in DMSO was in the same orientation. Regardless of this, hydrogen bonds were not frequently observed to explain this fact. However, AV and DT also generated docking solutions with the quinolinyl group of bedaquiline and imidazolyl in miconazole in same position, highlighting the capacity of the employed algorithms. From the docking solutions of our hit list, that of miconazole was the most similar to X77 in the crystal structure, in other words, the imidazole ring in S5, the nonpolar substituted phenyl in S2 and the six-membered ring at the boundary between S3 and S4 (Figure 8), supporting the hypothesis of interactions between the main protease and this drug.

Although no compound had a mean binding affinity value statistically lower than that of X77 or had improved complementarity to the SARS-CoV-2 main protease, two approved oral drugs and one approved buccal drug presented comparable performance to a representant of the micromolar inhibitor class. Also, it worth mentioning the scaffold diversity observed in the hit list, in other words, compounds with linear shapes and very polar moieties (glibenclamide), with a very rigid cross shape and many aromatic groups (bedaquiline), or lipophilic with considerable flexibility (miconazole). To the best of our knowledge, none of these three approved drugs have been suggested by any other previous VS study to target the SARS-CoV-2 main protease to date.

## Conclusion

The objective of this repurposing work was to select drugs employing two-step hierarchical virtual screening to suggest them as possible SARS-CoV-2 main protease (M^pro^) inhibitors for COVID-19 treatment. The framework relied on 3D LB and SB methods that allowed easy interpretation and more retention of information. As result, two oral and one buccal drugs from different classes and chemical characteristics were identified as promising M^pro^ inhibitors: bedaquiline, a very lipophilic antibiotic used for multi-drug resistant tuberculosis, glibenclamide, a second-generation sulphonylurea for diabetes mellitus type 2 treatment, and miconazole, an azole prescribed for the treatment of fungal infections. Many available crystal M^pro^ structures in complex with different inhibitors were successfully employed in the drug selection and docking analysis, also the analysis of these structures provides insights into future design studies for novel selective inhibitors of this protease.

## Future perspective

First reported in December 2019, the COVID-19 (coronavirus disease 2019) pandemic has had catastrophic effects on social, economic and public health in many countries around the world. In this context, it is crucial that new strategies are used to reduce viral proliferation and mortality.

This work indicates three possible noncovalent SARS-CoV-2 M^pro^ inhibitors that can be tested and, if active, used in therapy more quickly. In addition, the selected structures and the ligand/target interactions identified by our study provide important information for future design studies of new selective inhibitors of this protease.

**Summary points**

- A database of 3981 approved drugs was submitted to virtual screening analyses as a way to identify possible candidates for drug repurposing focusing on SARS-CoV-2 M^pro^.
- Three molecules (bedaquiline, miconazole and glibenclamide) were selected as possible noncovalent M^pro^ inhibitors based on characteristics of the Protein Data Bank code 6w63 crystal ligand (X77) and its orthostatic site.
- The predicted binding affinities of these three approved drugs were comparable to a known inhibitor.
- This novel information provides structural insights into developing more selective SARS-CoV-2 M^pro^ inhibitors and lead us to which classes of drugs and molecules can be repurposed to fight the current pandemic.
